# Glycemic Associations With Endothelial Function and Biomarkers Among 5 Ethnic Groups: The Multi‐Ethnic Study of Atherosclerosis and the Mediators of Atherosclerosis in South Asians Living in America Studies

**DOI:** 10.1161/JAHA.112.004283

**Published:** 2013-02-22

**Authors:** Alka M. Kanaya, Devon A. Dobrosielski, Peter Ganz, Jennifer Creasman, Ritu Gupta, Vidya Nelacanti, Jens Vogel‐Claussen, David Herrington

**Affiliations:** 1 University of California, San Francisco, CA (A.M.K., P.G., J.C., R.G., V.N.); 2 Towson University, Towson, MD (D.A.D.); 3 Johns Hopkins University School of Medicine, Baltimore, MD (J.V.C.); 4 Wake Forest University Medical Center, Winston‐Salem, NC (D.H.)

**Keywords:** biomarkers, diabetes, endothelium, ethnicity, insulin resistance

## Abstract

**Background:**

The association of prediabetic states with endothelial dysfunction measured by flow‐mediated dilation (FMD) or endothelial biomarker levels remains controversial. We examined data from 5 ethnic groups to determine the association between glucose categories and FMD or endothelial biomarkers. We determined whether these associations vary by ethnic group or body mass index.

**Methods and Results:**

We used data from 3516 participants from 5 race/ethnic groups with brachial FMD, endothelial biomarkers, and glucose category (normal, impaired fasting glucose [IFG], and diabetes) measures. There were significant ethnic differences in FMD, biomarker levels, and the prevalence of IFG and diabetes. However, all 5 ethnic groups showed similar patterns of higher FMD for the IFG group compared with the normal glucose and diabetes groups, which was most significant among whites and Asian Indians. Associations between glucose categories and endothelial biomarkers were more uniform, with the IFG and diabetes groups having higher biomarker levels than the normal glucose group. These associations did not change with further adjustment for fasting insulin levels. Whites with normal BMI had higher FMD values with higher glucose levels, but those with BMI in the overweight or obese categories had the inverse association (*P* for interaction=0.01).

**Conclusions:**

The discordance of IFG being associated with higher FMD but more abnormal endothelial biomarker levels is a novel finding. This higher FMD for the IFG group was most notable in whites of normal BMI. The higher FMD among those with impaired fasting glucose may reflect differences in insulin signaling pathways between the endothelium and skeletal muscle.

## Introduction

Endothelial dysfunction is a well‐recognized consequence of diabetes that leads to both micro‐ and macrovascular disease complications.^1^ However, endothelial activation and dysfunction may precede diabetes onset and may provide a mechanism that promotes insulin resistance and glucose dysregulation resulting in diabetes.^2–4^ Likewise, cellular adhesion molecules produced by endothelial cells, such as E‐selectin and intercellular adhesion molecule (ICAM)–1, and other proteins expressed in endothelial cells and released with endothelial activation (such as von Willebrand factor [vWF] and plasminogen activator inhibitor [PAI]–1) are elevated in prediabetic individuals and also in those with diabetes.^5–8^

The prevalence of diabetes varies widely among different racial/ethnic groups**,** with most ethnic minority groups having higher rates of prediabetes and type 2 diabetes than European populations.^9–10^ However, the factors underlying these disparate diabetes rates are unclear.^11^ Mean levels of endothelial biomarker proteins^12–15^ and mean values of brachial artery flow‐mediated dilation (FMD), a noninvaisive measure of endothelial function**,**^16–18^ are also known to vary by race/ethnic group. FMD and endothelial protein markers may provide additional information about those at risk for diabetes. Yet it is unknown whether the association between FMD or endothelial biomarkers and glycemic status varies by ethnic group.

We compared the mean levels and adjusted associations between FMD and endothelial biomarkers with glucose categories (normal, impaired fasting glucose, and diabetes) among 5 ethnic groups. We also determined whether these associations varied among ethnic groups or by body mass index (BMI) and whether the associations could be explained by fasting insulin level. We hypothesized that FMD would be lower and endothelial biomarkers would be higher in a stepwise manner with higher glucose category, but that these associations would be similar in all ethnic groups.

## Methods

We used data from the baseline MESA examination (2000–2002) and the second MASALA study clinical visit (2009–2010) for these analyses. MESA includes individuals from 4 racial/ethnic groups (whites, African Americans, Latinos, and Chinese Americans), and MASALA includes only Asian Indians. The MASALA study was modeled on the Multi‐Ethnic Study of Atherosclerosis (MESA), using similar recruitment methods, eligibility criteria, questionnaire, clinical measurements, ultrasound reading center, and research laboratory for biomarker assays to make efficient cross‐ethnic comparisons.^11^

### MESA Study

The study design, eligibility**,** and methods for MESA have been previously published.^19^ Individuals with physician‐diagnosed heart attack, stroke, transient ischemic attack, congestive heart failure, angina**,** or atrial fibrillation on 12‐lead electrocardiogram and those using nitroglycerin were excluded. Individuals with a history of cardiovascular procedures such as coronary artery bypass graft surgery, angioplasty, valve replacement, pacemaker**,** or defibrillator implantation or of any surgery on the heart or arteries were also excluded. Persons under active cancer treatment, with impaired cognitive ability, with a life expectancy <5 years, or with plans to move out of the study region**,** living in a nursing home**,** or on a waiting list were also excluded.

The baseline MESA questionnaire included questions on sex, race/ethnicity, age, education, and smoking. Serum glucose was measured from fasting samples by the glucose oxidase method (Johnson & Johnson Clinical Diagnostics, Inc). Fasting serum insulin was measured by an immunoenzymatic sandwich assay using Access® Ultrasensitive Insulin Reagent Packs on the Access® Immunoassay System (Beckman Instruments, Inc). Hypertension was defined as self‐reported treatment for hypertension or a systolic blood pressure ≥140 mm Hg or diastolic blood pressure ≥90 mm Hg. Participant weight was measured on a standard balance‐beam scale, and height was measured using a stadiometer. Waist circumference was measured using a Gullick II tape spring‐tension measure at the site of maximum circumference midway between the lower ribs and the anterior superior iliac spine. The mean of 2 waist circumference measurements was calculated.

Of the 6814 MESA participants, 6489 (95.2%) underwent FMD testing. Participants were excluded from the FMD procedure if they had a history of Raynaud's phenomenon (n=55), a congenital abnormality of the arm or hand (n=12), or a radical mastectomy on either side (n=100). Participants with blood pressures in the left and right arms that differed by >15 mm Hg or had uncontrolled hypertension (systolic blood pressure >180 mm Hg or diastolic blood pressure >110 mm Hg) were also excluded from having the FMD procedure (n=158). Images from 5731 participants were of sufficient quality for reading, but because of financial constraints, images were read for only a subset of participants (n=3501). To have similar age range of participants from both studies, individuals in MESA ≥80 years old were excluded from the analysis (n=112).

### MASALA Study

The Metabolic syndrome and Atherosclerosis in South Asians Living in America (MASALA) study was conducted in 2006–2007. Participant eligibility and recruitment methods have been previously published.^11^ We invited the 150 enrolled participants for a second follow‐up visit (April 2009–January 2010).

Participants underwent anthropometric and seated blood pressure examinations by a trained research assistant, who performed all measurements using standardized MESA protocols as described above. Blood samples were obtained from all participants following a 12‐hour fast. Plasma glucose was measured using an automated analyzer with an immobilized enzyme biosensor (YSI 2300 STAT Plus, YSI Life Sciences, Yellow Springs, OH). Serum insulin was measured by radioimmunoassay (Millipore, St. Charles, MO).

Of the 150 participants enrolled in the MASALA study, 132 (88%) completed this second clinical examination. We excluded 4 participants who had poor‐quality brachial ultrasound readings and 1 participant with Raynaud's phenomenon, leaving a total of 127 Asian Indian individuals for this analysis.

### Predictor

We categorized individuals into 3 distinct glucose tolerance categories: normal, impaired fasting glucose (IFG), and type 2 diabetes. We used the American Diabetes Association^20^ criteria for diabetes, requiring that a participant be using a hypoglycemic medication or have a fasting glucose ≥126 mg/dL. Impaired fasting glucose was defined as fasting plasma glucose between 100 and 125 mg/dL.^21^ Normal glucose participants had a fasting glucose <100 mg/dL.

### Brachial FMD Methodology

Brachial FMD was assessed with ultrasound in the MESA and MASALA cohorts using identical protocols. The ultrasonagraphers for both studies were trained in the FMD protocol and were certified by MESA reading center personnel.

Participants were examined in the supine position following 15 minutes of rest and after at least a 6‐hour fast. An automated sphygmomanometer (Dinamap device) was used to monitor blood pressure in the left arm. The right arm was supported at the elbow and wrist, and an occlusive blood pressure cuff was placed around the right forearm ≈2 inches below the antecubital fossa. The right brachial artery was imaged 5 to 9 cm above the antecubital fossa using a 9‐MHz linear‐array multifrequency transducer. Prior to cuff inflation, 30 seconds of baseline brachial ultrasound data were collected. To document the vasodilator response, the brachial artery was imaged a second time beginning immediately prior to cuff deflation and extending for 2 minutes after cuff release.

The videotaped data were analyzed at the Wake Forest University Cardiology Image Processing Laboratory with a previously validated semiautomated system.^22^ The semiautomated readings (media‐adventitial interfaces to media‐adventitial interfaces) of these digitized images generated the baseline and maximum diameters of the brachial artery from which %FMD was computed as follows: %FMD=([maximum diameter−baseline diameter]/baseline diameter)×100%. Intrareader and intrasubject variability from the MESA study has previously been reported,^23^ and a single reader interpreted all the MASALA study scans.

### Endothelial Biomarker Outcomes

The 4 endothelial protein biomarkers for both studies were assayed by the same research laboratory (Laboratory for Clinical Biochemistry Research University of Vermont) using identical assays and methods. We measured E‐selectin and soluble intercellular adhesion molecule–1 (sICAM‐1) from frozen EDTA‐plasma specimens and plasminogen activator inhibitor–1 (PAI‐1) and percent von Willebrand Factor (vWF) from citrated plasma specimens. Soluble E‐selectin and sICAM‐1 were both measured by ELISA (R&D systems, Minneapolis, MN), with an interassay CV range of 3.1% to 5.3% for E‐selectin and 3.3% to 6.4% for sICAM‐1. PAI‐1 was measured by ELISA (Diagnostica Stago, Inc, Parsippany, NJ), with an interassay CV of 2.9% to 8.2%. Percent vWF was measured by immunoturbidmetric methods by the STA‐R analyzer, with an interassay CV of 4.4% to 7.5%.

### Statistical Analysis

Both MESA and MASALA participant data were combined to examine racial/ethnic differences in the relationship between glucose categories and FMD or endothelial markers. We evaluated racial differences in 8 outcome measures including 4 brachial artery ultrasound variables and 4 endothelial activation biomarkers using ANOVA. Brachial artery ultrasound outcomes included baseline diameter (in millimeters) of the artery before the inflation of the blood pressure cuff, the maximum diameter (in millimeters) observed during hyperemic blood flow, the absolute change in FMD, and percent change in FMD. All outcome measures were examined as continuous variables. Normal probability plots of the residuals were used to detect violations of the normality assumption.

To test whether race/ethnicity modified the association between glucose category and the above outcomes, a race‐by‐glucose interaction term was including in the multivariable regressions model. Separate multivariable linear regression models were used to estimate the association between glucose category with the brachial ultrasound outcomes and endothelial protein markers within each ethnic/racial group. All models were adjusted for age, sex, education, hypertension, and BMI. Models for absolute and percent change in FMD also included baseline brachial artery diameter. If associated with the outcome (*P*<0.2) after adjustment, models also included use of statins (PAI‐1 and vWF models) or of angiotensin‐converting enzyme inhibitors (ACEIs) or angiotensin receptor blockers (ARBs) for the PAI‐1 and E‐selectin models. To show these associations graphically for each outcome, we calculated adjusted means with 95% CIs for each glucose category by race/ethnic group and among all participants combined. In a final model, we also adjusted for fasting insulin levels to determine whether the observed associations would be attenuated. We checked whether BMI category would modify the association between FMD or biomarkers and glucose category. Significant interactions were added to the above multivariate model.

To explore the potential for nonlinear associations, we modeled the percent change in FMD and fasting glucose using cubic splines in an adjusted generalized additive model. We also modeled the percent change in FMD by fasting glucose with adjusted cubic splines for standard BMI categories (normal, overweight, and obese) among whites. Smoothed plots of the predictor variable by partial residuals were plotted to show the relationship between the predictor and outcome after adjusting for potentially confounding variables. Additional sensitivity analyses were conducted to measure the influence of extreme values. All analyses were conducted in SAS version 9.2 (SAS Institute, Cary, NC).

## Results

Data from 3389 MESA participants and 127 MASALA participants were available for this analysis. Table 1 displays the demographic and anthropometric data and clinical status for participants from each of the 5 ethnic groups. Mean age was 60.7±9.4 years, and approximately half the participants were women. There were significant differences in educational attainment by ethnicity, with Asian Indians having the highest proportion with bachelor's degree or higher (80%) and Latinos having the lowest (11%). Asian Indians and Chinese Americans were more likely to be never smokers (83% and 76%, respectively), and African Americans had the highest proportion of current smokers (19%). BMI varied by ethnic group, with the highest BMI among African Americans (29.6±5.5 kg/m^2^) and lowest for Chinese Americans (24.0±3.2 kg/m^2^). Glucose categories also varied by ethnicity, with Asian Indians having the highest prevalence of diabetes (DM) and IFG (24% and 19%, respectively), followed by Latinos (16% for both DM and IFG), African Americans (15% DM and 14% IFG), Chinese Americans (13% DM and 17% IFG), and whites (5% DM and 9% IFG). Statin and ACEI/ARB use was highest among Asian Indians and lowest among Chinese Americans.

**Table 1.** Characteristics of the MESA (2000–2002) and MASALA (2009–2010) Participants

Variable	Overall (n=3516)	White (n=1131)	Chinese American (n=613)	African American (n=766)	Latino (n=879)	Asian Indian (n=127)
Age, mean±SD	60.7±9.4	61.0±9.3	60.8±9.6	60.9±9.4	60.0±9.5	59.5±8.1
Female sex	1761 (50)	557 (49)	305 (50)	387 (51)	451 (51)	61 (48)
Highest educational attainment						
≤High school	1251 (36)	221 (20)	234 (38)	225 (29)	558 (63)	13 (10)
Some college/associates or tech degree	949 (27)	291 (26)	129 (21)	286 (37)	231 (26)	12 (9)
Bachelor's degree	635 (18)	279 (25)	140 (23)	133 (17)	50 (6)	33 (26)
Graduate degree	681 (19)	340 (30)	110 (18)	122 (16)	40 (5)	69 (54)
Family income ≥$75 000	863 (25)	445 (40)	119 (20)	139 (19)	65 (8)	95 (76)
Currently drinks alcohol	1734 (49)	758 (67)	128 (21)	387 (51)	396 (45)	65 (51)
Smoking status						
Never smoker	1897 (54)	499 (44)	466 (76)	346 (45)	481 (55)	105 (83)
Past smoker	1192 (34)	505 (45)	114 (19)	274 (36)	282 (32)	17 (13)
Current smoker	427 (12)	127 (11)	33 (5)	146 (19)	116 (13)	5 (4)
BMI (kg/m^2^), mean±SD	27.9±5.2	27.8±5.0	24.0±3.2	29.6±5.5	29.3±5.1	26.4±4.6
Waist circumference (cm), mean±SD	97.2±14.1	98.5±14.5	86.9±9.7	100.7±14.3	100.1±12.8	94.4±12.2
Fasting glucose, mean±SD	96.9±29.1	90.5±20.0	99.1±29.8	98.2±29.3	101.8±36.5	101.6±26.0
Serum insulin (mU/L), mean±SD	10.6±13.6	9.6±6.1	9.6±13.7	11.2±23.4	11.1±8.3	17.0±8.6
Hypertension	1597 (45)	467 (41)	240 (39)	452 (59)	383 (44)	55 (43)
Glycemic status						
Normal	2602 (74)	961 (85)	432 (71)	538 (70)	601 (68)	70 (57)
Impaired fasting glucose	479 (14)	106 (9)	103 (17)	108 (14)	139 (16)	23 (19)
Diabetes	423 (12)	60 (5)	77 (13)	118 (15)	138 (16)	30 (24)
Statin medication use	493 (14)	172 (15)	76 (12)	102 (13)	111 (13)	32 (25)
ACEI or ARB use	407 (12)	114 (10)	33 (5)	115 (15)	116 (13)	29 (23)

MESA indicates Multi‐Ethnic Study of Atherosclerosis; MASALA, Mediators of Atherosclerosis in South Asians Living in America; SD, standard deviation; BMI, body mass index; ACEI, angiotensin‐converting enzyme inhibitor; ARB, angiotensin receptor blocker.

### Comparison of Brachial Ultrasound Outcomes and Endothelial Biomarker Levels by Race/Ethnic Groups

Baseline brachial artery diameter differed significantly by race/ethnicity (*P*<0.001) and was smallest for Asian Indians (4.1±0.8 mm) and largest for African Americans (4.5±0.8 mm) (Table 2). Absolute change in brachial artery diameter with FMD differed among the 5 ethnic groups, and percent change in FMD was greatest for whites and Chinese Americans (4.8±3.0% and 4.8±2.7%, respectively) and lowest for African Americans (3.6±2.6%). Unadjusted E‐selectin, sICAM, and vWF levels also varied by ethnic group (Table 2). Latinos had the highest and Asian Indians the lowest levels of E‐selectin (61.9 versus 42.1 ng/mL, respectively) and sICAM‐1 (288.3 ng/mL for Latinos versus 213.3 ng/mL for Asian Indians). PAI‐1 levels were similar across all race/ethnic groups. Percent vWF was highest among African Americans (149±60%) and lowest among Asian Indians (124±47%).

**Table 2.** Endothelial Function and Biomarkers by Race/Ethnic Group (Mean±SD)

	Overall (n=3516)	White (n=1131)	Chinese American (n=613)	African American (n=766)	Latino (n=879)	Asian Indian (n=127)	*P*‐Value*
Brachial artery ultrasound measures							
Baseline diameter, mm	4.3±0.8	4.2±0.8	4.3±0.7	4.5±0.8	4.4±0.8	4.1±0.8	<0.001
Postdeflation diameter, mm	4.5±0.8	4.4±0.8	4.5±0.7	4.6±0.8	4.6±0.8	4.2±0.8	<0.001
Absolute change in diameter, mm	0.2±0.1	0.2±0.1	0.2±0.1	0.2±0.1	0.2±0.1	0.2±0.1	<0.001
FMD, % change in diameter	4.4±2.8	4.8±3.0	4.8±2.7	3.6±2.6	4.4±2.7	4.1±2.7	<0.001
Endothelial markers							
E‐selectin, ng/mL	51.8±23.7	48.4±20.0	52.3±27.9	55.2±23.2	61.9±27.9	42.1±16.4	<0.001
sICAM, ng/mL	267.4±77.6	283.2±66.4	228.9±57.7	261.3±91.2	288.3±86.8	213.3±58.4	<0.001
PAI‐1 (ng/mL)	26.9±27.1	27.2±31.8	28.6±19.6	24.2±23.5	27.9±28.0	26.3±20.0	0.55
von Willebrand factor, %	133.4±53.8	131.1±53.4	132.8±53.2	149.1±60.3	133.6±52.6	124.0±47.4	0.01

SD indicates standard deviation; FMD, flow‐mediated dilation; sICAM, soluble intercellular adhesion molecule; PAI‐1, plasminogen activator inhibitor 1.

* *P* for comparison among the 5 ethnic groups.

### Associations Between FMD and Endothelial Biomarkers and Glucose Groups

We found a significant interaction between race/ethnicity and glucose category for several outcomes (*P* for interaction=0.04 for absolute and percent change in FMD, *P*=0.01 for E‐selectin, and *P*=0.007 for sICAM). Therefore, all further analyses are presented both overall and by individual racial/ethnic category.

**Figure 1.**
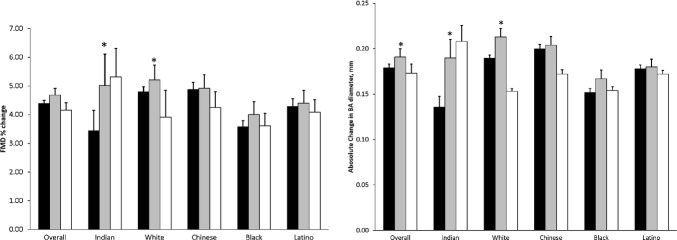
Adjusted mean FMD values by glucose category and ethnic group (adjusted for age, sex, BMI, education, hypertension, and baseline BA diameter, *P<0.05; black bar, normal glucose; gray bar, impaired fasting glucose; white bar, diabetes group). FMD indicates flow‐mediated dilation; BMI, body mass index; BA, brachial artery.

Adjusted means for the 2 FMD outcomes by ethnic group and glucose category are shown in Figure 1. Among all participants combined, there appeared to be an inverted U‐shaped association, with the IFG group having the highest absolute and percent change in FMD (ie, better endothelial function) compared with the normal and diabetes groups, which had relatively lower levels of FMD (*P* for heterogeneity=0.01). This inverted U‐shaped association was significant in whites (*P* for heterogeneity=0.001) but not in the other ethnic groups. However, Asian Indians had a different pattern for FMD, with higher FMD levels associated with higher glucose category (*P*=0.002). Further adjustment for fasting serum insulin levels did not change these significant associations.

**Figure 2.**
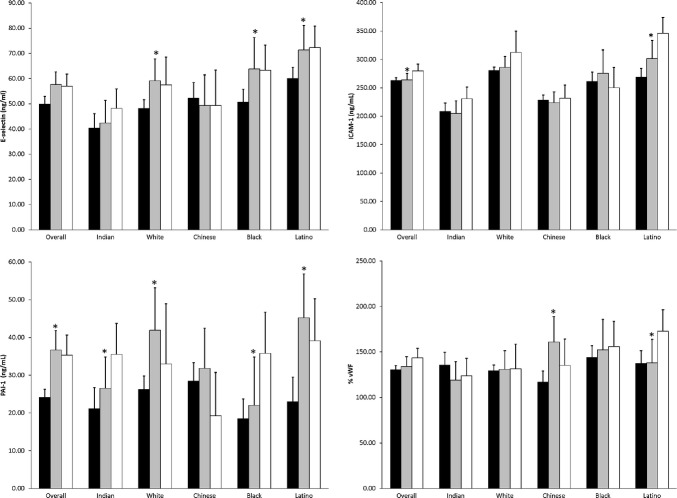
Adjusted associations between glucose categories and endothelial markers by ethnic group (*P<0.05). ICAM‐1 indicates intercellular adhesion molecule 1; PAI‐1, plasminogen activator inhibitor 1; vWF, von Willebrand factor.

Adjusted mean levels of endothelial biomarkers were somewhat more uniform in association with glucose categories (Figure 2). Higher levels of sICAM‐1 and PAI‐1 were associated with higher glucose category among all participants. Whites, African Americans, and Latinos had similar increased levels of E‐selectin for the IFG and diabetes groups compared with the normal glucose group. However, the adjusted association between PAI‐1 level and glucose category had an inverted U‐shaped association similar to the FMD outcomes, with the IFG groups having somewhat higher levels than those with diabetes and much higher levels than the normal glucose group. This pattern was significant among whites and Latinos (*P*<0.001 for each), whereas for the African American and Asian Indian participants, PAI‐1 levels monotonically increased with increasing glucose category. There was a significant association for vWF with glucose category observed among Chinese Americans and Latinos only. Again, the addition of fasting insulin did not change the observed associations.

**Figure 3.**
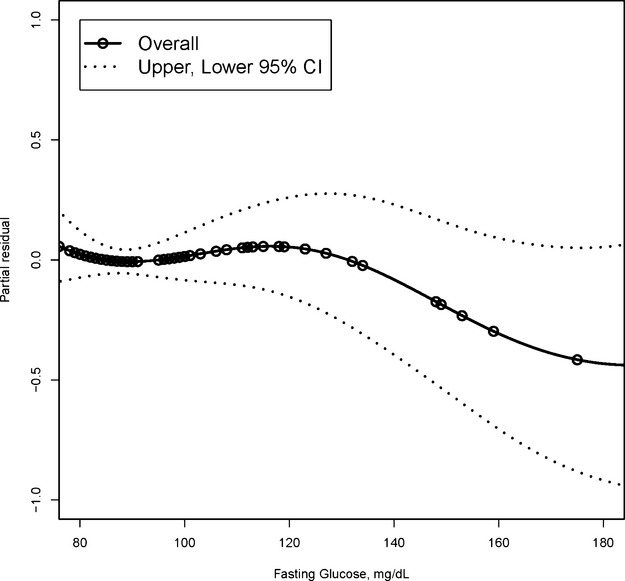
Adjusted cubic splines showing percent change in FMD by fasting glucose level for all 5 race/ethnic groups combined. Each symbol represents 1 observation from a random sample of 100 observations. FMD indicates flow‐mediated dilation; CI, confidence interval.

**Figure 4.**
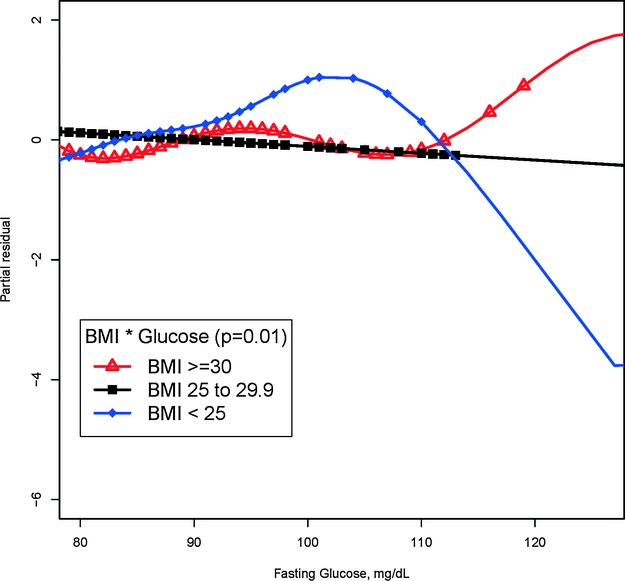
Adjusted cubic splines showing percent change in FMD by fasting glucose among whites by BMI category. Each symbol represents 1 observation from a random sample of 100 observations per group. FMD indicates flow‐mediated dilation; BMI, body mass index.

Finally, we examined the percent change in FMD outcome by continuous fasting glucose levels in all 5 ethnic groups using adjusted cubic splines (Figure 3). The splines showed a curvilinear association for FMD with increasing glucose level, with higher FMD in the prediabetes glucose range (100 to 126 mg/dL) and lower FMD in the diabetes range of glucose values. When we checked for interaction by BMI category in post hoc analyses, we found that whites with normal BMI had higher FMD values, with higher glucose values ranging from 80 to 110 mg/dL, but those whose BMI was in the overweight or obese category had the inverse association, with lower FMD with higher glucose values (*P* for interaction=0.01, Figure 4). Sensitivity analyses excluding extreme values yielded similar results.

## Discussion

Among these middle‐aged community‐based populations, we found ethnic differences in brachial artery flow‐mediated dilation, most endothelial biomarker levels, and the prevalence of impaired fasting glucose and diabetes. There were similar trends in the association between FMD and glucose categories for the MESA ethnic groups, with the IFG group having higher FMD than the other 2 glucose groups, which was not explained by fasting insulin levels. This association was most significant among whites with normal BMI. The pattern was somewhat different among Asian Indians, who had higher FMD for the IFG and diabetes groups compared with the normal glucose group, not explained by medication use, insulin levels, or BMI. There were more consistent relationships observed between glucose categories and endothelial biomarkers among all ethnic groups, with the impaired fasting group and diabetes groups having higher biomarker levels than the normal glucose group.

Several prior studies have examined the association between glucose categories and endothelial function by FMD, yielding inconsistent findings. In the large Dutch Hoorn study, the diabetes group had lower FMD percent change than the normal glucose group, but there was no difference in FMD for those in the intermediate glucose category (composed of those with either or both impaired fasting glucose and impaired glucose tolerance) compared with those with normal glucose.^24^ In the Northern Manhattan Study, higher fasting blood glucose level was associated with lower levels of FMD, and those with prediabetes had a 25% decrease in FMD compared with participants with normal glucose.^25^ Although the Northern Manhattan study was performed in a multiethnic population, the results were not separated by race/ethnic group to determine whether this association was similar in all groups. In a recent Greek study, among those without diabetes or symptomatic cardiovascular disease, HbA1c level was inversely associated with FMD, but this was only significant among those with low BMI.^26^Other investigators have studied the effect of acute hyperglycemia on endothelial function and found no association.^27–28^ Our study found lower FMD as expected among those with diabetes compared with those with normal glucose for the 4 MESA ethnic groups. However, we observed an unexpectedly higher FMD among those with IFG compared with the other glucose categories among all 5 ethnic groups, which was most significant among whites with normal BMI and in the relatively small Asian Indian sample. Further adjustment for statins or other vasodilating medications such as ACE inhibitors and ARBs also did not explain this effect. We hypothesized that higher circulating insulin levels in the IFG state may explain this effect, but adjusting for insulin did not attenuate this finding. The consistency of the elevated FMD in the impaired fasting glucose group among all 5 ethnic groups in adjusted models makes this finding more compelling but needs to be confirmed by other large studies.

Other studies have found similarly higher levels of endothelial markers among individuals with prediabetes or diabetes compared with normal glucose levels.^5,7^ In a prospective analysis from the Nurses Health Study, Meigs and colleagues found that E‐selectin and ICAM‐1 were independently associated with incidence of diabetes.^6^ The biologic mechanisms determining endothelial protein secretion differ from the mechanisms for flow‐mediated dilation, which may account for the differing results for the impaired fasting glucose groups.

Although we had data from 5 ethnic groups to compare for this analysis, we were limited by small numbers of Asian Indians in the MASALA study. The higher FMD levels observed among Asian Indians with diabetes compared with the other 2 groups may be spurious, as the confidence intervals for the mean FMD levels per glucose group were large.

Although all of the 5 ethnic groups had trends showing higher FMD for the IFG group compared with the other 2 glucose groups, this finding was only significant in whites (with the lowest prevalence of IFG) and Asian Indians (with the highest prevalence of IFG), not in the 3 other ethnic groups with intermediate IFG prevalence. Although we found that circulating levels of serum insulin did not attenuate this finding, there are known differences in the insulin signaling pathway in the endothelium compared with that in skeletal muscle.^29–31^ If the response to insulin is relatively maintained by the endothelium in the face of skeletal insulin resistance among individuals with IFG, the higher concentrations of circulating insulin may enhance vasodilatory effects and improve FMD. In addition, the IFG category represents a heterogeneous group with variable defects in first‐phase insulin secretion, insulin resistance, and hepatic gluconeogenesis, and the prevalence of IFG varies by ethnic group.^32^ Interestingly, this association between IFG and higher FMD was observed in whites with normal BMI <25 kg/m^2^, which is consistent with the modest BMI levels among the Asian Indians in the MASALA study. It is possible that the mechanisms for IFG are similar among whites and Asian Indians because of closer genetic ancestry in these 2 racial/ethnic groups compared with the other 3 ethnic groups in MESA.^33^ This finding of greater FMD among those with IFG compared with those with diabetes may be related to the findings of many large epidemiologic studies that have found no increase in cardiovascular disease outcomes among the IFG category.^34^

In conclusion, we found that individuals with impaired fasting glucose had higher FMD levels than those with diabetes or normal glucose, which was not explained by fasting insulin levels, whereas endothelial biomarker levels were higher among those with IFG and diabetes compared with those with normal glucose. The increased FMD among those with impaired fasting glucose may reflect known differences in insulin signaling pathways between the endothelium and skeletal muscle.^29^ This finding may explain why there is no consistent increased cardiovascular disease risk among those in the IFG group.
